# Molecular Engineering of Functional SiRNA Agents

**DOI:** 10.1021/acssynbio.4c00181

**Published:** 2024-05-11

**Authors:** Neelu Batra, Mei-Juan Tu, Ai-Ming Yu

**Affiliations:** Department of Biochemistry and Molecular Medicine, UC Davis School of Medicine, Sacramento, California 95817, United States

**Keywords:** RNA engineering, small interfering RNA, RNA interference, therapy

## Abstract

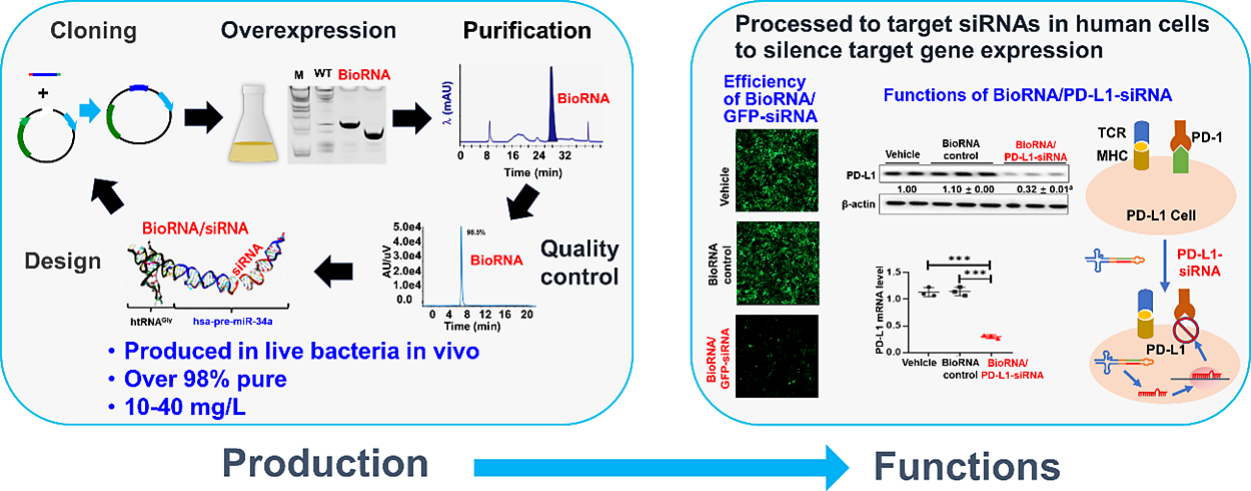

Synthetic biology constitutes a scientific domain focused on intentional redesign of organisms to confer novel functionalities or create new products through strategic engineering of their genetic makeup. Leveraging the inherent capabilities of nature, one may address challenges across diverse sectors including medicine. Inspired by this concept, we have developed an innovative bioengineering platform, enabling high-yield and large-scale production of biological small interfering RNA (BioRNA/siRNA) agents via bacterial fermentation. Herein, we show that with the use of a new tRNA fused pre-miRNA carrier, we can produce various forms of BioRNA/siRNA agents within living host cells. We report a high-level overexpression of nine target BioRNA/siRNA molecules at 100% success rate, yielding 3–10 mg of BioRNA/siRNA per 0.25 L of bacterial culture with high purity (>98%) and low endotoxin (<5 EU/μg RNA). Furthermore, we demonstrate that three representative BioRNA/siRNAs against GFP, BCL2, and PD-L1 are biologically active and can specifically and efficiently silence their respective targets with the potential to effectively produce downstream antiproliferation effects by PD-L1-siRNA. With these promising results, we aim to advance the field of synthetic biology by offering a novel platform to bioengineer functional siRNA agents for research and drug development.

## Introduction

Synthetic biology entails the engineering of novel biological entities to generate relevant agents including production of medical biomolecules.^1^ In various industries, researchers have successfully developed and commercialized numerous biologically relevant agents through the implementation of synthetic biology.^1^ One of the latest fields to use synthetic biology is the study and application of RNA interference (RNAi).^2^ RNAi is a prominent means of modulating gene expression and a natural defense mechanism in some eukaryotic cells, allowing for direct regulation of gene expression.^3,4^ This regulatory mechanism has been harnessed in the field of drug development, specifically in the creation of RNAi-based therapies.^5−9^ These therapies include the use of small interfering RNAs (siRNAs) that have the potential to revolutionize the way that diseases are treated by specifically and selectively silencing a gene of interest to treat the underlying causes of diseases at the genetic level. As a result, the US Food and Drug Administration (FDA) has granted approval to six siRNA drugs, Patisiran (Onpattro, 2018), Givosiran (Givlaari, 2019), Lumasiran (Oxlumo, 2020), Inclisiran (Leqvio, 2021), Vutrisiran (Amvuttra, 2022), and Nesodiran (Rivfloza, 2023),^5,8−10^ while others undergo clinical and preclinical trials,^8,11−16^ with many more expected to follow in the coming years.^9,17^

**Figure 1:**
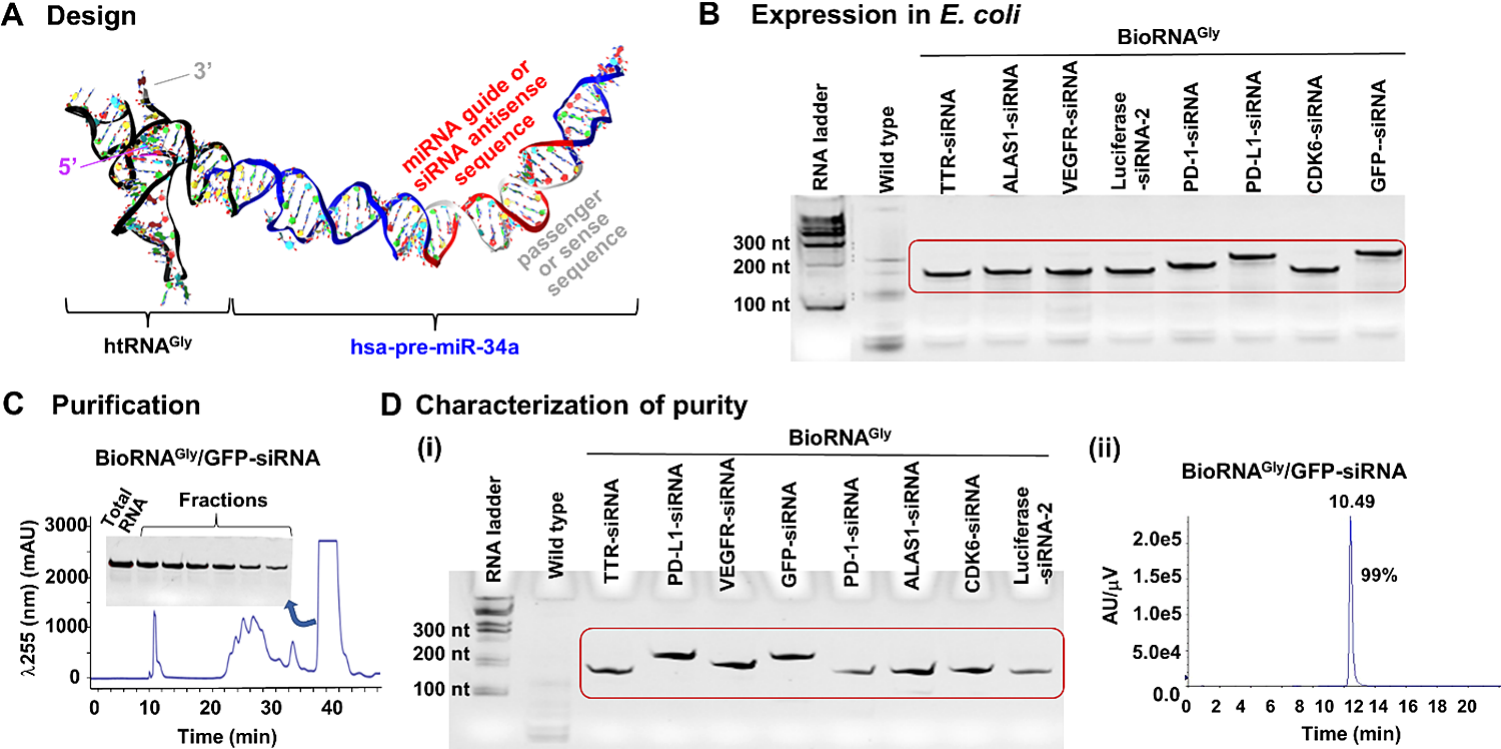
(A) Predicted 3D structural illustration of the recombinant biological siRNA (BioRNA/siRNA) using a human glycyl tRNA fused pre-miR-34a carrier. (B) Overexpression of target BioRNA/siRNAs is confirmed by urea-PAGE analysis of total RNAs extracted from bacteria transformed with individual BioRNA expression plasmids. Wild-type bacterial total RNA was used for comparison. (C) Representative FPLC-UV trace during the purification of model BioRNA^Gly^/GFP-siRNA and urea-PAGE analysis of isolated BioRNA fractions compared with total RNA. (D) Purities of engineered siRNA molecules were (i) semiquantitatively determined by urea-PAGE analysis and (ii) quantitatively by HPLC analyses, as modeled by BioRNAGly/GFP-siRNA. Endotoxin levels were also measured and are depicted in Table 1.

Today, RNAi-based research and therapeutics primarily rely on the utilization of chemically engineered or in vitro synthesized RNA agents.^18,19^ However, there is an increasing concern regarding the use of chemically modified RNA as the introduction of artificial elements may alter the physical and chemical properties, structures, or activities and thereby may induce unknown side effects or immunogenicity.^20−23^ In other words, these alterations may potentially lead to differences in effectiveness and safety not observed in naturally synthesized and modified RNA equivalents. Driven by the desire to create more natural RNA molecules for research and therapy purposes and to better harness the structures and properties of endogenous RNA molecules, we have recently developed a groundbreaking technology based on the tRNA fused pre-miRNA carriers (Figure 1A) that enables high-yield, highly homogeneous, and large-scale production of a wide range of bioengineered RNA agents (BioRNAs) within living host cells.^24,25^

This carrier has proven effective in expressing various types of small RNA molecules, including siRNA, miRNA, and RNA aptamers.^26−28^ Leveraging this carrier, our recent studies have validated our novel platform for bioengineering small RNA molecules, allowing for large-scale production through bacterial fermentation. Moreover, our previous research demonstrated that BioRNA agents showcased selective and efficient release of the target small RNA within human cell lines and animal models.^26−31^ Importantly, our studies revealed that BioRNAs derived from this platform contained only a few, natural post-transcriptional modifications.^26,32^ Additionally, they exhibited favorable stability within human cells and patient-derived mouse models, highlighting their potential for therapeutic applications.^26,32^ In the present study, we aim to utilize our innovative bioengineering platform to create a panel of remarkably pure, homogeneous functional siRNA agents using a new human glycyl tRNA fused hsa-pre-miR-34a carrier (Figure 1A). Significantly, we will test the biological activity of three model siRNA agents and their abilities to specifically and substantially modulate their targets, consequently leading to a notable antitumor effect downstream using an in vitro engineered model.

## Results and Discussion

### Design, Overexpression, and Purification of Recombinant siRNA Molecules

*Escherichia coli* are the predominant organismal platform for expressing and producing recombinant proteins.^33^ As such, we suspected that *E. coli* would demonstrate similar efficacy for RNA expression and production to satisfy the growing demand for RNA-based drugs. Therefore, our previous works endeavored to optimize a bacterial fermentation platform to produce small RNA molecules for research and therapeutics,^27−29,34^ while the mail goal of our current study aims to produce functional siRNA agents via bacterial fermentation for research and therapeutics. To achieve this, our RNA bioengineering platform uses a recently designed and established human tRNA, Glycine-GCC (BioRNA^Gly^), fused with an optimized pre-miR-34a sequence^27^ (Figure 1A) to develop a stable small RNA carrier.^31^ Basically, this stems from novel discovery demonstrating the consistent and stable expression with significant accumulation of specific tRNA/pre-miRNA molecules within bacterial systems.^26,28,32,35^ In principle, a tRNA/pre-miRNA complex could function as a stable carrier for noncoding RNA (ncRNA), and new constructs can be generated by replacing the miRNA duplex sequence of the pre-miRNA with target siRNA sequences. In our current study, we used this carrier to design a panel of nine BioRNA/siRNA agents by independently substituting the miR-34a duplex of the pre-miR-34a sequence with each siRNA sequence of interest (Figure 1A). Among the panel are two siRNA therapeutics already approved by the FDA [siRNA against transthyretin (TTR-siRNA); siRNA against 5-aminolevulinic acid synthase (ALAS1-siRNA)], four potential candidate siRNAs in preclinical or clinical trials [siRNA against vascular endothelial growth factor receptor (VEGFR-siRNA), an siRNA against programmed cell death protein 1 (PD-1-siRNA), an siRNA against programmed cell death ligand 1 (PD-L1-siRNA), an siRNA against B-cell lymphoma 2 (BCL2-siRNA)],^36−48^ and three siRNAs routinely used in basic research [siRNA against green fluorescent protein (GFP-siRNA); siRNA against luciferase (Luciferase-siRNA); and siRNA against cell division protein kinase 6 (CDK6-siRNA)].^49−56^

To achieve overexpression, each BioRNA/siRNA (Table 1) was cloned into a pBSTNAV vector as previously described^27,28^ using specific primers (Supplementary Table S2). The recombinant plasmids thus produced were verified by DNA sequencing and were transformed to HST08 *E. coli* cells for overnight small-scale fermentation (15 mL). Total RNAs extracted from small-scale fermentation were analyzed by urea-PAGE to confirm the overexpression of individual BioRNA/siRNAs, as indicated by the presence of a new, prominent band accounting for over 50% of total RNAs at expected size (∼200 nt) with a 100% success rate, compared to wild-type bacterial total RNA (Figure 1B).

**Table 1:** Yields, Purities, and Endotoxin Levels of BioRNA/siRNA Agents Produced Using Humanized tRNA^Gly^/pre-miR-34a Carriers, Isolated by the Anion Exchange FPLC Method

**BioRNA**^**Gly**^	**yield (mg RNA/0.25 L fermentation)**	**purity (%; by HPLC)**	**endotoxin level (EU/μg RNA)**
TTR-siRNA	4.50	98.8	2.83
ALAS1-siRNA	6.30	98.9	0.75
VEGFR-siRNA	6.00	99.4	1.40
Luciferase-siRNA	10.6	99.2	4.76
PD-1-siRNA	3.30	99.2	2.27
PD-L1-siRNA	4.70	99.1	2.40
CDK6-siRNA	7.20	99.4	1.00
GFP-siRNA	5.26	99.4	4.30

To generate and purify large quantities of BioRNA/siRNA agents for functional studies, we extracted the total RNA from large-scale fermentation (0.25 L) of HST08 *E. coli* cells transformed with respective BioRNA/siRNA-expressing plasmids. To purify our BioRNA/siRNA agents, 5–6 mg of extracted total RNA was separated on an ENrich-Q 10 × 100 column using an optimized anion exchange fast protein liquid chromatography (FPLC) method as previously described^27,28^ (Figure 1C) and fractions were collected of the overexpressed peak. Each collected fraction was then run on a urea-PAGE gel along with the respective total RNA to verify their homogeneity (Figure 1C) where only the fractions containing single bands were pooled, concentrated, and desalted using centrifugal filter units. The purities of final concentrated siRNA products were semiquantitatively determined by urea-PAGE analysis (Figure 1D. i) and quantitatively by high-performance liquid chromatography (HPLC) analyses, as modeled by BioRNAGly/GFP-siRNA (Figure 1D. ii). Quantitative analysis of HPLC purity and assessment of endotoxin levels by the LAL endotoxin assay kit demonstrated that all BioRNA/siRNAs were produced with very high purity (>98%) and exhibited low endotoxin activity (<5 EU/μg RNA) (Table 1). Table 1 also summarizes the yield of all the purified BioRNA/siRNAs produced per 0.25 L fermentation to be in the range of 3–10 mg with low endotoxin levels, suggesting a high-level expression and a high-quality BioRNA/siRNA product thus produced by our fermentation technology.

### BioRNA/GFP-siRNA Is Effective in Knocking Down GFP Expression in Multiple Cancer Types

**Figure 2:**
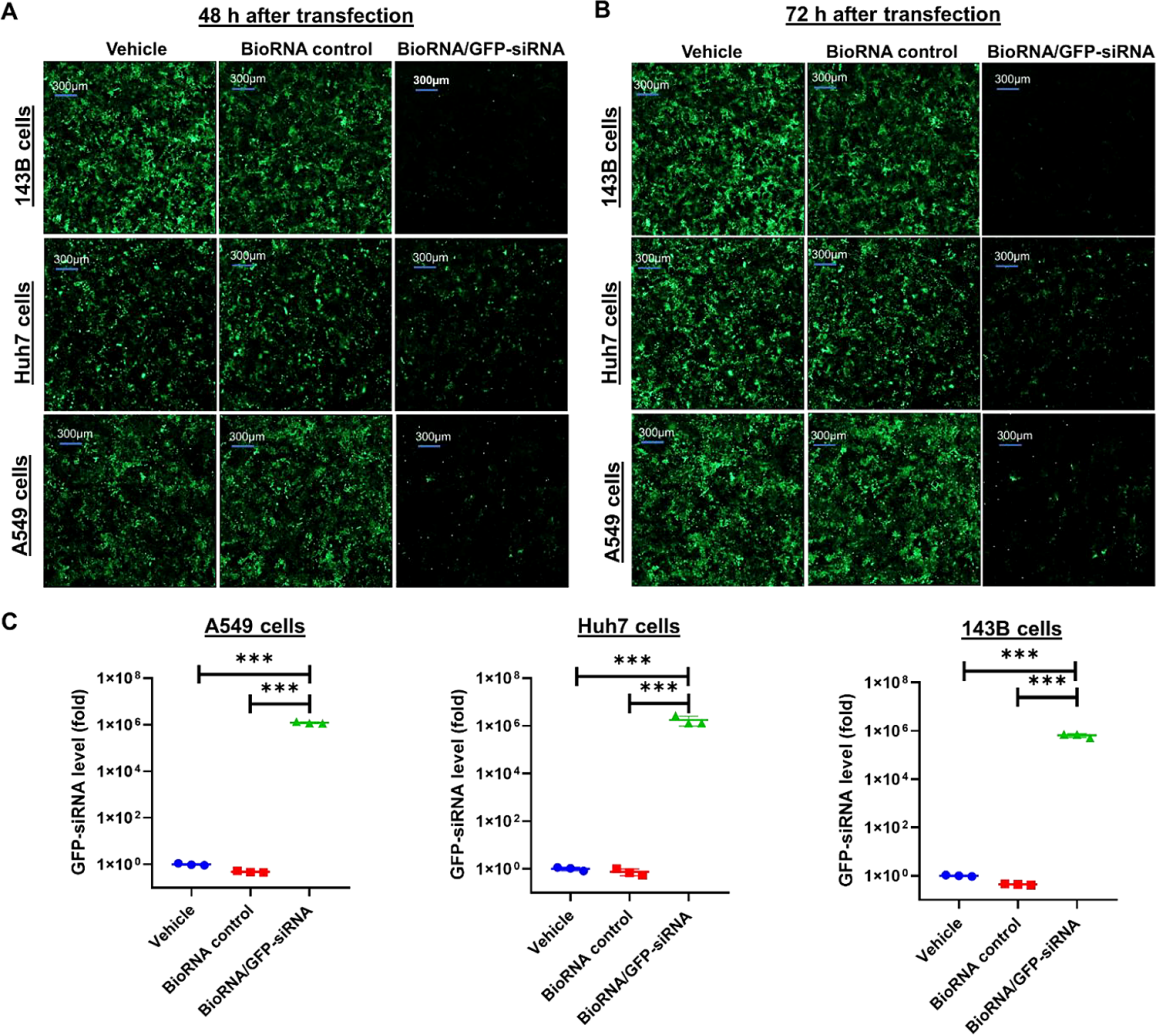
GFP fluorescence intensities were sharply reduced by recombinant GFP-siRNA in multiple human cell lines at 48 h (A) and 72 h (B) post-transfection. GFP-expressing A549, Huh7, and 143B cells were treated with 15 nM BioRNA/GFP-siRNA, control RNA, or vehicle alone (Lipofectamine 3000), and images were acquired by using ImageXpress Pico. (C) BioRNA/GFP-siRNA was processed to target GFP-siRNA in individual cell lines. Cells were treated with 15 nM of BioRNA/GFP-siRNA, control BioRNA, or vehicle for 48 h, and GFP-siRNA levels were determined by the selective stem-loop RT-qPCR assay. Values are the mean ± SD (*N* = 3/group). *P < 0.05, **P < 0.01, and ***P < 0.001 (one-way ANOVA with Bonferroni *post hoc* tests).

To further assess the functionality of our BioRNA/siRNA agents, we selected three model BioRNA/siRNA agents (BioRNA/GFP-siRNA, /BCL2-siRNA, and/PD-L1-siRNA) to test their efficacy in different cell models. To evaluate the efficacy of our model BioRNA/GFP-siRNA agent, we tested its capability to knock down GFP in three GFP-overexpressing cell models from different cancer types, lung cancer (A549-GFP-Luc cells), hepatic cancer (Huh7-GFP-Luc cells), and osteosarcoma (143B-GFP-Luc cells) at 24, 48, and 72 h post-transfection. Compared to the controls, our results describe a remarkable reduction of GFP fluorescence intensity in all cell lines starting 24 h post-transfection (Supplementary Figure S1) with further reduction observed at 48 and 72 h (Figure 2A,B). These results indicate that the model BioRNA/GFP-siRNA agent is highly effective in downregulating its specific target protein, GFP. However, to ensure that BioRNA/GFP-siRNA is indeed effectively processed to mature GFP-siRNA in all three cell types, we employed a selective stem-loop reverse transcription quantitative real-time PCR (RT-qPCR) method to evaluate the siRNA recovered from cells transfected with 15 nM BioRNA/GFP-siRNA and controls after 48 h. These data showed significantly higher levels of mature GFP-siRNA levels as compared to controls in all the cell types (Figure 2C).

Together, these results demonstrate that the model BioRNA/GFP-siRNA was processed to mature GFP-siRNA which was functionally active in multiple cancer types by downregulating target GFP expression as early as 24 h post-transfection. Further, the downregulation of target GFP expression was also observed to increase with time as shown at 48 h and even more at 72 h post-transfection, demonstrating the in vitro stability of our BioRNA/siRNA agent.

### BioRNA/BCL2-siRNA Reduces the Target Gene Expression in Human Cells

**Figure 3:**
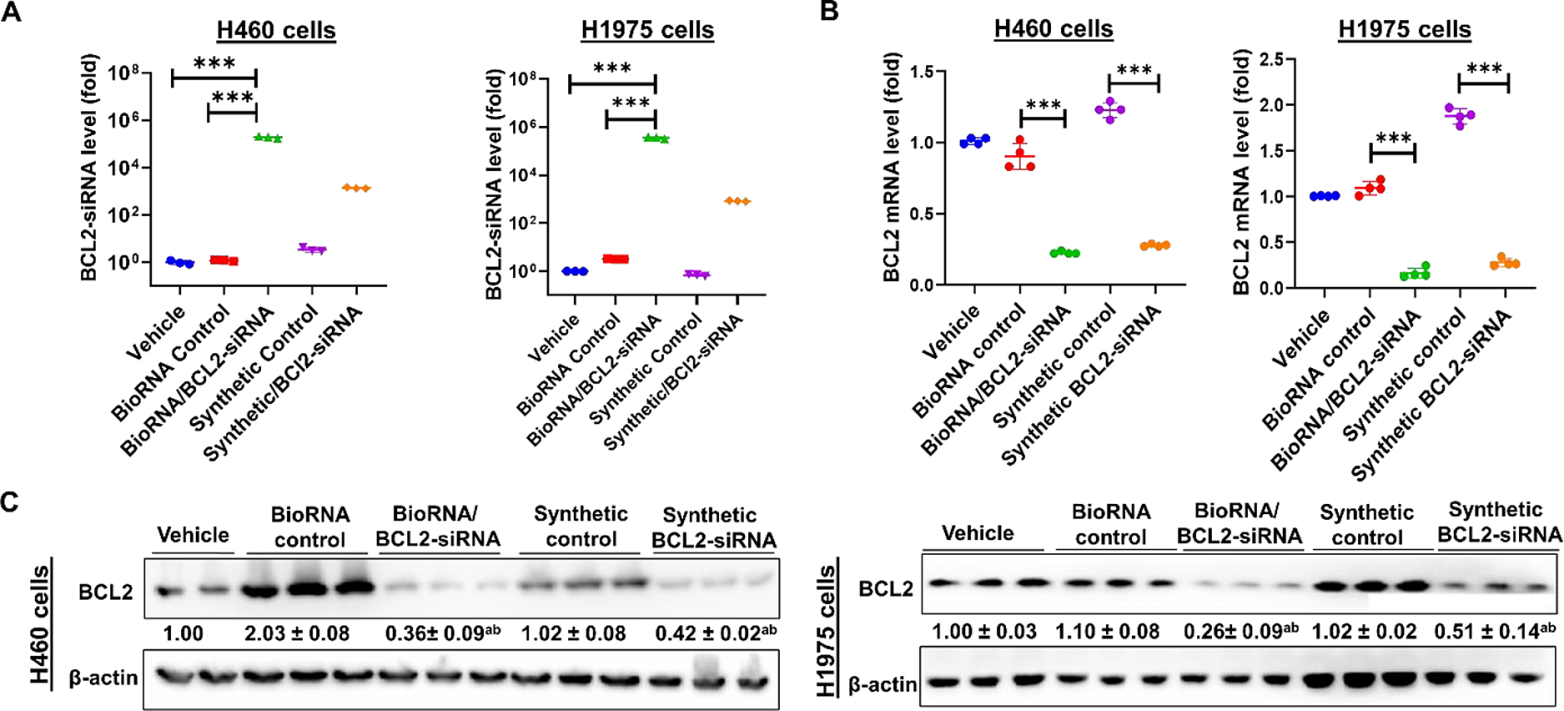
Both BioRNA/BCL2-siRNA and its synthetic counterpart siRNA were processed to their mature forms (A), as determined by the selective stem-loop RT-qPCR assay, and were effective in modulating target mRNA levels (B), as determined by selective RT-qPCR assays, and target gene expression (C), as determined by immunoblot analysis in lung carcinoma cells (H1975 and H460). The cells were treated with 15 nM of BioRNA/BCL2-siRNA, its synthetic counterpart, synthetic control RNA, control BioRNA, and vehicle alone (Lipofectamine 3000) for 48 h for siRNA/mRNA quantitation and 72 h for immunoblot analyses. Values are the mean ± SD (*N* = 3/group). *P < 0.05,**P < 0.01, and ***P < 0.001 (one-way ANOVA with Bonferroni *post hoc* tests). Band intensity for immunoblot analysis was normalized to the corresponding β-actin level. ^a^*P* <0.05 compared to vehicle treatment, and ^b^*P* <0.05 compared to respective control RNA; 1-way ANOVA with Bonferroni post-tests.

We next selected model BioRNA/BCL2-siRNA to test its functionality and to compare its efficacy with chemically synthesized siRNA, having the same sequence purchased from a known vendor, in multiple nonsmall cell lung cancer (NSCLC) cells. To ensure the effective processing of BioRNA/BCL2-siRNA to mature BCL2-siRNA, we used a selective stem-loop RT-qPCR method to determine the levels of mature BCL2 following treatment with 15 nM of BioRNA/BCL2-siRNA, synthetic/BCL2-siRNA, and respective controls for 48 h in H460 and H1975 cells. Next, to compare the effectiveness of BioRNA/BCL2-siRNA and synthetic/BCL2-siRNA to regulate the expression of their target at the mRNA and protein level in NSCLC cells, we performed RT-qPCR and immunoblot analyses. Our data demonstrated the successful processing of our BioRNA/BCL2-siRNA agent to its mature form (Figure 3A) and a significant downregulation of BCL2 mRNA (approximately 70–80%) and protein (approximately 60–75%) as compared to the controls in both H460 and H1975 cells (Figure 3B,C).

Surprisingly, BioRNA/BCL2-siRNA resulted in a significantly higher level of mature BCL2-siRNA than the chemically synthesized siRNAs with equivalent transfection concentrations (Figure 3A). Also, as shown in Figure 3C, the BioRNA/BCL2-siRNA demonstrated superior efficacy in the downregulation of target BCL2 in H1975 cells as compared to the synthetically produced respective siRNA, whereas the downregulation of target protein by BioRNA/BCL2-siRNA in H460 was comparable to the synthetic commercial product.

### BioRNA/PD-L1-siRNA Is Effective in Downregulating Human PD-L1 and Activated Immune Cells for Antitumor Activity

**Figure 4:**
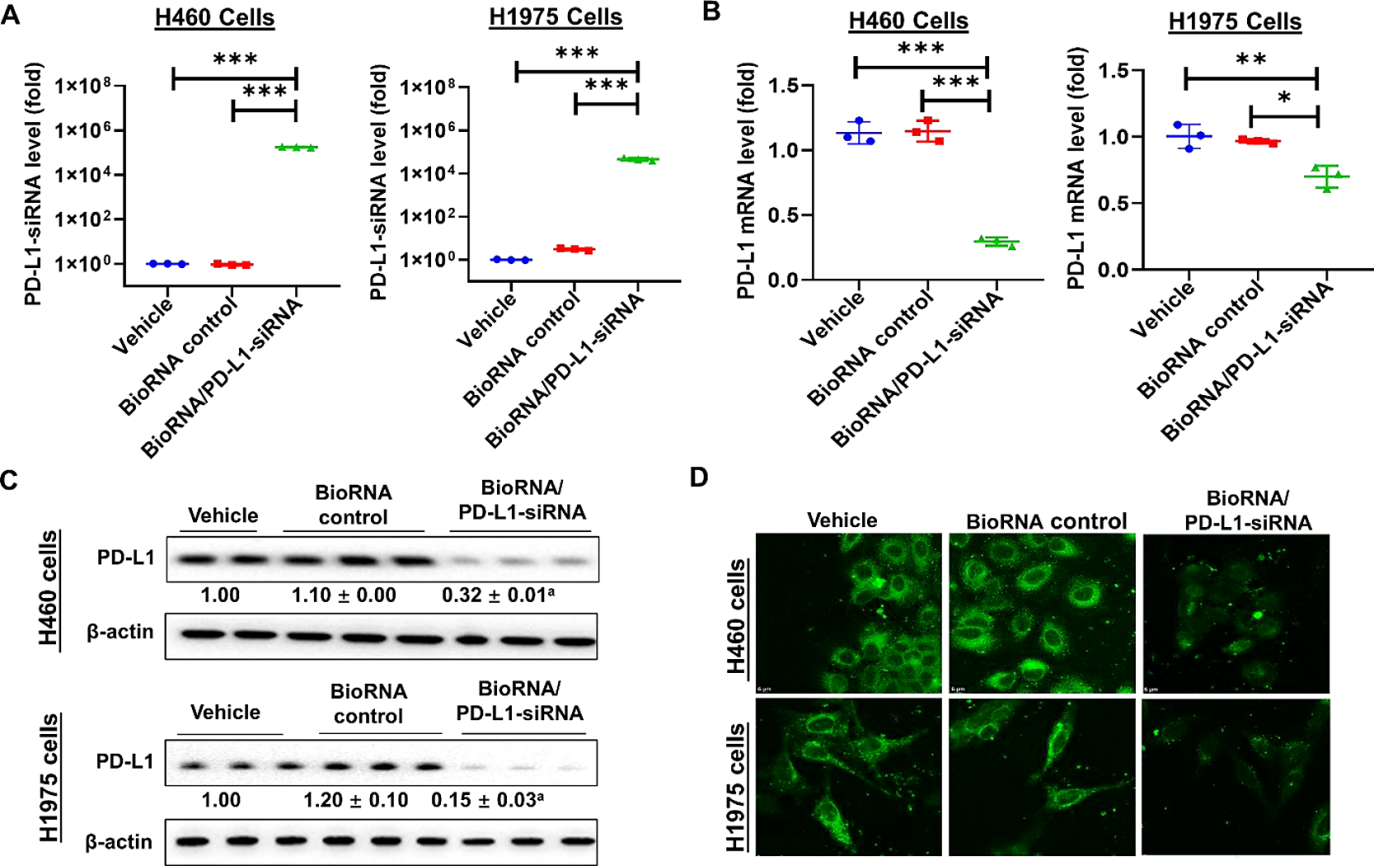
BioRNA/PD-L1-siRNA was processed to mature PD-L1-siRNA to effectively reduce PD-L1 mRNA and protein levels in both H460 and H1975 cells, as demonstrated by (A, B) selective RT-qPCR, (C) Western blot, and (D) IF analyses. Values are the mean ± SD (*N* = 3/group). ^a^*P* < 0.05, compared to control RNA; *P < 0.05,**P < 0.01,and ***P < 0.001 (one-way ANOVA with Bonferroni *post hoc* tests).

To assess the functionality of our third model agent, BioRNA/PD-L1-siRNA, we employed multiple biochemical analyses in NCSLC cells and bioengineered Chinese hamster ovary (CHO) cells. First, we used a selective stem-loop RT-qPCR method to ensure the processing of BioRNA/PD-L1-siRNA to mature PD-L1-siRNA following treatment with 15 nM of BioRNA/PD-L1-siRNA and controls for 48 h in H460 and H1975 cells. Compared to the controls, our results demonstrate significantly higher levels of PD-L1-siRNA confirming the successful processing of our siRNA agent in two different NSCLC cells (Figure 4A). To assess the effectiveness of BioRNA/PD-L1-siRNA in regulating the expression of its target at the mRNA and protein levels in NSCLC cells, we employed RT-qPCR and immunoblot analyses. Our data showed that as compared to the controls, there was significant downregulation of the target mRNA levels (approximately 30–60%, Figure 4B) and target protein levels (approximately 70–80%, Figure 4C) in both H460 and H1975 cells. We further carried out immunofluorescence studies to determine the impact of BioRNA/PD-L1-siRNA on PD-L1 protein expression in the same two NSCLC cells. Our immunofluorescent data confirmed the plasma membrane localization of PD-L1 and demonstrated remarkable reduction of PD-L1 protein in both cell lines (Figure 4D) as compared to the controls.

**Figure 5:**
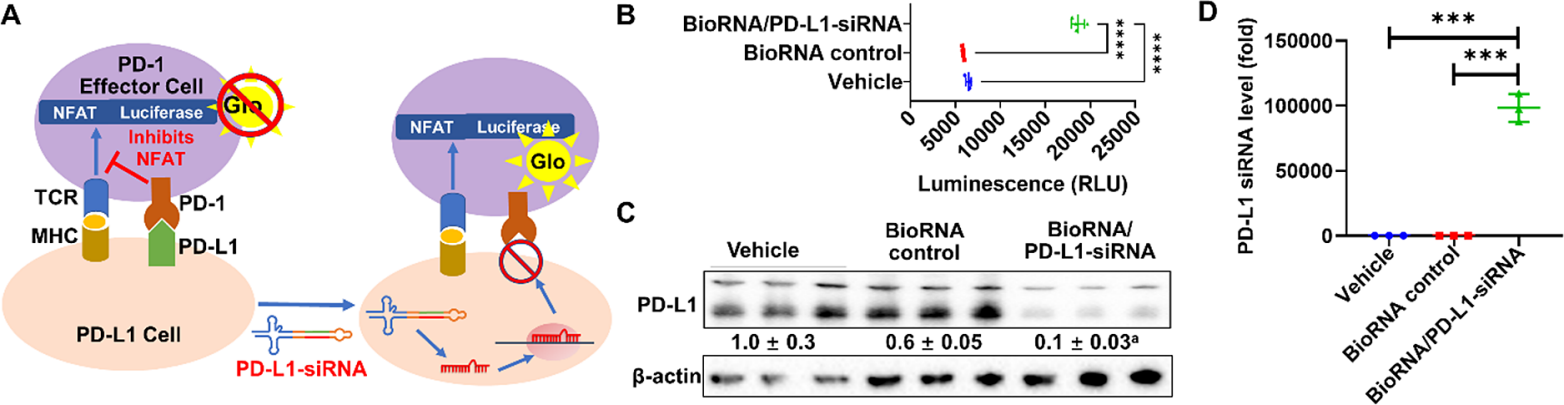
(A) Illustration of the PD-1/PD-L1 blockade assay depicting the disruption of PD-1/PD-L1 interaction elevating luminescent reporter activity. (B) Treatment with BioRNA/PD-L1-siRNA disrupted the PD-1/PD-L1 interaction, as demonstrated by a significant increase in luminescence signal. (C) Reduction of the PD-L1 protein level by BioRNA/PD-L1-siRNA in PD-L1 overexpressing CHO cells was confirmed by Western blot analysis, which was associated with the (D) significantly increased level of PD-L1-siRNA, as determined by the selective stem-loop RT-qPCR method. Values are the mean ± SD (*N* = 3/group). ^a^*P* < 0.05, compared to control RNA; ***P < 0.001, and ****P < 0.0001 (one-way ANOVA with Bonferroni *post hoc* tests).

To assess if our siRNA agents can not only downregulate their specific target but also induce downstream regulatory activity, we employed our model BioRNA/PD-L1-siRNA in a reporter gene bioassay purchased from Promega (WI, USA) that is designed to test the potency and stability of biologics designed to block the PD-1/PD-L1 interaction. This assay was used to measure the potency and stability of BioRNA/PD-L1-siRNA to interfere with the PD-1/PD-L1 interaction. This PD-1/PD-L1 block bioassay utilizes two genetically engineered cell lines. The first is the Jurkat T cell line that stably expresses human PD-1 and nuclear factor of activated T-cells (NFAT) to induce luciferase. The second is the CHO-K1 cell line that stably expresses human PD-L1, and a cell surface protein designed to activate cognate T cell receptors (TCRs) in an antigen-independent manner. In principle, when cocultured, the PD-1/PD-L1 interaction inhibits the TCR signaling and NFAT-mediated luciferase activity or TCR-mediated luminescence (Glo) (Figure 5A). However, when transfected with BioRNA/PD-L1-siRNA, the expression level of PD-L1 is reduced, thus releasing the inhibitory signal that results in TCR signaling and NFAT-mediated luciferase activity causing luminescence (Glo) that can be detected with a luminometer (Figure 5A).

To test the potency of our BioRNA/PD-L1 siRNA, we employed this bioassay and our results showed that not only can our BioRNA/PD-L1-siRNA be additionally processed to mature PD-L1-siRNA in the engineered CHO cells (Figure 5D) but can also efficiently downregulate human PD-L1 on the surface that reduced the binding of human PD-1 on the surface of T cells and transmitted the activation signal of T cells (Figure 5B). The immunoblot analysis of the treated CHO cells also further validated the PD-1/PD-L1 block bioassay, as clearly demonstrated by a significant downregulation of PD-L1 as compared to the controls (Figure 5C). These results indicated that BioRNA/PD-L1-siRNA downregulation of PD-L1 in return released the immune inhibition of the PD1/PD-L1 axis, thus activating immune effector cells for putative downstream antitumor activities. Overall, through this study, we have demonstrated that our bioengineering platform produces high-quality and biologically functional siRNA agents capable of specifically and significantly modulating the respective target gene expressions and may efficiently result in downstream antitumor effects.

## Conclusions

The RNAi therapy has emerged as a promising treatment option for several previously untreatable diseases, demonstrating significant potential across a wide spectrum of diseases such as rare genetic diseases, cardiovascular diseases, cancer, viral infections, diabetes, and numerous others. The FDA approvals and ongoing clinical trials across various stages highlight the promising future of this rapidly evolving field. In this study, we have used a new human tRNA fused hsa-pre-miR-34a carrier-based bioengineering platform to produce a panel of novel high yielding, highly homogeneous, and functional BioRNA/siRNA agents via bacterial fermentation for basic research and therapeutic studies. Processed within live cells and bearing only natural post-transcriptional modifications, BioRNA/siRNA agents are distinguished from existing siRNA entities in commercial or clinical use today. Our findings also show that BioRNA/siRNA agents thus produced can be processed to target siRNAs that are biologically active and potent in regulating target gene expression in human cells.

With our novel bioengineering approach and through the establishment of an exclusive platform enabling the economical and sustainable production of natural siRNA agents within living cells, we introduce a groundbreaking class of siRNA agents that offer a more reflective representation of the physicochemical, biological, and safety attributes observed in natural RNAs. These novel siRNA agents hold tremendous potential for both fundamental and applied research as they facilitate the production of natural and cost-effective siRNA agents. This advancement not only will amplify the functional genomics research capabilities but also will stimulate the advancement of RNA-based therapeutics. The implications are expected to be far-reaching, encompassing a wide spectrum of diseases and expanding the potential druggable targets across various domains of synthetic biology.

## Materials and Methods

### Chemicals and Materials

RPMI 1640 medium, phosphate-buffered saline (PBS), 0.05% Trypsin-EDTA, fetal bovine serum (FBS), Opti-MEM, bicinchoninic acid (BCA) protein assay kit, Lipofectamine 3000, *E. coli* strain DH5α, and radioimmunoprecipitation assay (RIPA) buffer were purchased from Thermo Fisher Scientific (Waltham, MA, USA), and *E. coli* strain HST08 and the cloning kit were purchased from Clontech Laboratories (Mountain View, CA). Protease inhibitor cocktail and Trizol reagent were purchased from Sigma-Aldrich (St. Louis, MO, USA). Bovine serum albumin (BSA), dimethyl sulfoxide (DMSO), and 2 × YT media were bought from VWR (Radnor, PA, USA). Western ECL Chemiluminescence Substrate, Blotting-grade Blocker, PVDF membrane, and TGX Stain-Free Fast Cast Acrylamide kit (10%) were purchased from Bio-Rad (Hercules, CA, USA). Direct-zol RNA miniprep kit was purchased from Zymo Research (Irvine, CA, USA). Primers used in this study were purchased from Integrated DNA Technologies (Coralville, IA). The synthetic siRNA negative control (Cat #AM4613) and the synthetic BCL2-siRNA (Sense 5′GGATGCCTTTGTGGAACTGTA3′, Antisense 5′TACAGTTCCACAAAGGCATCC3′) were purchased from Thermo Fisher Scientific. All other chemicals and organic solvents of analytical grade were purchased from Thermo Fisher Scientific, VWR, or Sigma- Aldrich.

### Cell Culture

Human carcinoma cell lines NCI-H460 (H460, HTB-177), A549 (CCL-185), and NCI-H1975 (CRL-5908) were purchased from American Type Culture Collection (Manassas, VA). The GFP and Luciferase-expressing A549-GFP-Luc, Huh7-GFP-Luc, and 143B-GFP-Luc cells were generated and reported previously^57−59^ after transduction with pCCLc-Luc-EGFP lentiviral constructs (Vector Core, UC Davis Medical center, Sacramento, CA). All cell lines were maintained in either RPMI 1640 or DMEM supplemented with 10% fetal bovine serum and grown in a 37 °C incubator maintained in a humidified atmosphere with 5% CO_2_. PD-L1 aAPC/CHO-K1 cells (Promega, Cat #J1252, Madison, WI, USA) were cultured in 90% Ham’s F12 (Gibco Cat #11765–054) with 10% FBS, 200 μg/mL hygromycin B, and 200 μg/mL G418 sulfate solution. PD-1 effector cells (Promega, Cat #J1250) were cultured in 90% RPMI-1640, supplemented with 10% FBS, 200 μg/mL hygromycin B, 500 μg/mL G418 sulfate solution, 1% sodium pyruvate, and 1% MEM nonessential amino acids.

### Plasmid Construction

The bioengineering of siRNA molecules was conducted with the same strategy as previously described with some minor optimizations.^57,60^ The siRNA sequences used in this study are listed in Table S1. For the plasmid construction, the inserts with coding target siRNA sequences were produced by PCR amplification. We used specific glycine tRNA primers with the respective template plasmids having the desired siRNA sequence for amplification of insert. Next, in-fusion cloning technology was used for amplicon ligation into the pBSTNAV plasmid vector according to the manufacturer's protocols, which was further transformed into *E. coli* HST08 competent cells. About 4–5 individual colonies were picked for each construct for small-scale fermentation (15 mL), and the plasmids isolated were then sent for DNA sequencing (Azenta, South San Francisco, US).

### In Vivo Heterologous Expression of Recombinant BioRNA/siRNA in *E. coli* and Purification of Target RNAs

Fermentation of *E. coli* transformed with a respective sequence-confirmed plasmid was performed in 2× YT media in a round-bottom 2 L flask on a large scale (0.25 L) and incubated at 37 °C on a shaker for 16 h, as described recently.^31,57,60^ Total RNAs were isolated from the bacterial pellets using the Tris-HCl-saturated phenol extraction method, and target RNA overexpression was verified by conducting the denaturing urea (8 M) polyacrylamide (8%) gel electrophoresis. The ethidium bromide-stained urea-PAGE gels were then imaged using a Chemi-Doc MP Imaging System (Bio-Rad, CA, USA).

All the target BioRNA/siRNAs and the BioRNA control used in this study were purified using an NGC Quest 10 Plus FPLC Chromatography system (Bio-Rad) as we previously described.^31,57,60^ The mobile phases of buffer A (10 mM NaH_2_PO_4_, pH 7.0) and buffer B (10 mM NaH_2_PO_4_, 1 M NaCl, pH 7.0) were used for the purification. All the buffers were made using autoclaved DEPC-treated H_2_O. The FPLC schematic flow was performed using the flow rate of 2 mL/min and started with 100% buffer A for 0–5 min; 55% buffer B for 5–10 min; a gradient 55–75% buffer B for 10–40 min followed by wash cycles. Following the purification, the purity was quantitated by the HPLC method optimized on a Shimadzu LC-20AD HPLC system with an XBridge Oligonucleotide BEH C18 column (2.1 × 50 mm, 2.5 μm particle size; Waters, Milford, MA, USA), also as previously described.^31,57,60^ All of the purified target BioRNAs were tested for endotoxin levels by the Pyrogent-5000 kinetic LAL assay (Lonza, Walkersville, MD).

### Knockdown of GFP in Human Cells

The 143B-GFP-Luc, Huh7-GFP-Luc, and A549-GFP-Luc cells were seeded on a 6-well plate (200,000 cells/well) that was compatible with the imager and transfected with vehicle, 15 nM of control RNA, or BioRNA/GFP-siRNA. The fluorescence was monitored with an ImageXpress Pico Automated Cell Imaging System by molecular devices at 24, 48, and 72 h post-transfection. All images were acquired under the same settings and at the same time for all of the treatment groups.

### RNA Extraction and RT-qPCR

Human NSCLC H460 and H1975 cell lines were seeded at a density of 200,000 cells/well in 6-well plates and incubated overnight at 37 °C with 5% CO_2_, and next day, they were transfected with 15 nM respective RNA agents for 48 h using Lipofectamine 3000. The Direct-zol RNA Miniprep Kit was used to isolate total RNA which was then quantified with a Spark microplate reader (Männedorf, Switzerland). Next, cDNA was generated from the 300–500 ng of total RNA using NxGen M-MuLV reverse transcriptase (Lucigen, Middleton, WI, USA) and random hexamer primers or siRNA-specific stem-loop primers (Supplementary Table S3). A CFX96 Touch real-time PCR system (Bio-Rad) was used to perform Real-time qPCR using gene-specific primers (Supplementary Table S3) with iTaq Universal SYBR Green Supermix according to the manufacturer’s instructions. The siRNA and the mRNA levels were normalized to U6 snRNA and 18S rRNA in corresponding samples, respectively, and normalized to vehicle control group, using the formula 2^–ΔΔCT^, in which ΔCT = CT (target gene) – CT (18S or U6) and ΔΔCT = ΔCT (treatment group) – ΔCT (vehicle control).

### Protein Extraction and Immunoblot Analysis

The H460 and H1975 cells were seeded into 6-well plates at a density of 250,000 cells/well and treated with 15 nM respective RNAs for 72 h using Lipofectamine 3000. Next, the cells were harvested and lysed in RIPA buffer supplemented with a protease inhibitor cocktail for 30 min on ice. Protein concentrations were determined using a BCA Protein Assay Kit. Total proteins were separated on a 10% TGX Stain-Free SDS-PAGE gel, transferred onto a polyvinylidene difluoride (PVDF) membrane using the semidry Trans-Blot Turbo Transfer System (Bio-Rad), and the membranes were immediately imaged for total protein with a Chemi-Doc MP Imaging System (Bio-Rad). The membranes were then blocked in 5% milk for 1 h and were incubated with selective primary antibodies against target proteins including PD-L1 (1:1000; Cell Signaling Technology Cat #13684, Danvers, MA), BCL2 (1:1000; Cell Signaling Technology Cat #4223), and β-actin (1:5000 dilution; Sigma-Aldrich Cat #A5441), overnight at 4 °C. The next day, membranes were washed thrice before incubating with secondary horseradish peroxidase-labeled anti-rabbit IgG (1:10,000; Jackson Immuno-Research, West Grove, Pennsylvania, USA) or antimouse (1:3000 dilution; Cell Signaling Technology Cat #7076,) antibodies for 2 h at room temperature and washed thrice. The membranes were then incubated with the Clarity Western ECL substrates (Bio-Rad) according to the manufacturer’s instructions and imaged with the Chemi-Doc MP Imaging System (Bio-Rad).

### Immunofluorescence Analyses

H460 and H1975 cells were seeded (10,000 cells/well) in an 8-well chamber slide overnight for attachment. The following day, cells were treated with vehicle, 15 nM BioRNA/Control RNA, or BioRNA/PD-L1-siRNA. 48 h post-transfection, the medium was removed, and cells were washed twice with cold PBS and fixed with 4% paraformaldehyde for 20 min at 4 °C. Cells were then washed three times with PBS and blocked with 3% BSA in PBS for 30 min at room temperature and then incubated with selective anti-PD-L1 antibody (PD-L1 (Extracellular Domain Specific) (D8T4X) Rabbit mAb (Alexa Fluor 488 Conjugate, cell signaling)) in blocking buffer overnight at 4 °C. The following day, wells were washed three times in PBS and mounted using VECTASHIELD Antifade Mounting Medium (H-1000–10). Images were acquired through a confocal microscope, a Leica Stellaris 5 using a 63× oil objective.

### PD-1-PD-L1 Blockade Bioassay

The assay was carried out according to the manufacturer’s instructions (Promega, Cat #J1250). Briefly, PD-L1 aAPC/CHO-K1 cells were seeded at 30,000 cells/well at 200 μL of media in clear sterile 96-well plates followed by overnight incubation in a 37 °C incubator with 5% CO_2_. The next day, the medium was replaced, and the PD-L1 aAPC/CHO-K1 cells were treated with 15 nM RNA or vehicle and incubated for another 48 h. Following the incubation, the PD-1 effector cells (4 × 10^4^ cells/well) were added to the treated aAPC/CHO-K1 cells in 80 μL assay buffer (99% RPMI-1640 supplemented with l-glutamine +1% FBS) for 6 h. Bio-Glo Reagent (80 μL) was added next to each well, and the plate was incubated at ambient temperature for 20–30 min followed by transferring the supernatant to a white plate and reading the luminescence on a SpectraMax M3 microplate reader (Molecular Devices, Sunnyvale, CA, USA).

### Statistical Analysis

Values are the mean ± SD. Comparisons for different treatment groups were conducted by one-way ANOVA with Bonferroni *post hoc* tests (GraphPad Prism). The difference was considered statistically significant when the *P* value was less than 0.05 (*P* < 0.05).

## Supporting Information Available

GFP fluorescence intensities by recombinant GFP-siRNA in multiple cell lines after 24 h post-transfection; sequences of BioRNA and primers used for the construction of corresponding plasmids; primer sequences used for the construction of expression plasmids; and primer sequences used for stem loop RT-qPCR and RT-qPCR (PDF)
