# Supplementary material for: Molecular Engineering of Functional SiRNA Agents
Source: ACS Synth Biol. 2024 May 11;13(6):1906–15. doi: 10.1021/acssynbio.4c00181 (PMC11197084; doi:10.1021/acssynbio.4c00181)
Supplement: Supplementary file 1 — sb4c00181_si_001.pdf [file sb4c00181_si_001.pdf]

Supporting Information

Molecular Engineering of Functional SiRNA Agents

Neelu Batra, Mei-Juan Tu, Ai-Ming Yu*

Department of Biochemistry and Molecular Medicine, UC Davis School of Medicine, Sacramento, CA 95817, USA

*Address correspondence to: Dr. Ai-Ming Yu, Department of Biochemistry and Molecular Medicine, UC Davis School of Medicine, Sacramento, CA 95817, USA; Email: aimyu@ucdavis.edu.

Supplementary Table S1. Sequences of BioRNA and primers used for the construction of corresponding plasmids. Underlined sequences are tRNA scaffold, target siRNA sequences are in red and complementary sequences are in green.

BioRNA	Length (nt)	Sequence (5' to 3')	MW (Da)
**BioRNA^{Gly}**			
BCL2-siRNA	180	GCAUGGGUGGUUCAGUGGUAGAAUUCUCGCCUGGCCAGCUGUGAGUGUU UCUUUACAGUUCCACAAAGGCAUCCUUGUGAGCAAUAGUAAGGAAGGGAUG CCUUGUGGGAACUGUCUAGAAGUGCUGCACGUUGUUGGCCCACGCGGGAG GCCCGGGUUCGAUUCCCGGCCCAUGCACCA	58,399
PD-1-siRNA	180	GCAUGGGUGGUUCAGUGGUAGAAUUCUCGCCUGGCCAGCUGUGAGUGUUU CUUCUCUCUUUGAUCUGCGCCUUGUUGUGAGCAAUAGUAAGGAAGCAAGG CGCGAUACAAAGAGACUAGAAGUGCUGCACGUUGUUGGCCCACGCGGGAG GCCCGGGUUCGAUUCCCGGCCCAUGCACCA	58,219
PD-L1-siRNA	180	GCAUGGGUGGUUCAGUGGUAGAAUUCUCGCCUGGCCAGCUGUGAGUGUU UCUUAAUGCGUUCAGCAAAUGCCAGUUGUGAGCAAUAGUAAGGAAGCUGG CAUUGCUAGAACGCAUCUAGAAGUGCUGCACGUUGUUGGCCCACGCGGGA GGCCCGGGUUCGAUUCCCGGCCCAUGCACCA	58,266

CDK6-siRNA	180	GCAUGGGUGGUUCAGUGGUAGAAUUCUCGCCUGGCCAGCUGUGAGUGUU<br>UCUUUUCCUAGUUGAUCAACAUCUGUUGUGAGCAAUAGUAAGGAAGCAGG<br>UGUUAUCUAACUAGGACUAGAAGUGCUGCACGUUGUUGGCCCACGCGGGA<br>GGCCCGGGUUCGAUUCCCGGCCCAUGCACCA	58,167
GFP-siRNA	180	GCAUGGGUGGUUCAGUGGUAGAAUUCUCGCCUGGCCAGCUGUGAGUGUU<br>UCUUAGUUGUACUCCAGCUUGUGCCCUGUGAGCAAUAGUAAGGAAGGGCA<br>CAAGUGGUAGUACAACCUAGAAGUGCUGCACGUUGUUGGCCCACGCGGGA<br>GGCCCGGGUUCGAUUCCCGGCCCAUGCACCA	58,665
Luciferase-siRNA	180	GCAUGGGUGGUUCAGUGGUAGAAUUCUCGCCUGGCCAGCUGUGAGUGUU<br>UCUUUCGAAGUAUUCCGCGUACGUGUUGUGAGCAAUAGUAAGGAAGCACG<br>UACGGGACAUACUUCGCUAGAAGUGCUGCACGUUGUUGGCCCACGCGGGA<br>GGCCCGGGUUCGAUUCCCGGCCCAUGCACCA	58,235
VEGFR-siRNA	180	GCAUGGGUGGUUCAGUGGUAGAAUUCUCGCCUGGCCAGCUGUGAGUGUUU<br>CUUCUUACAGUUUUGUUUUUCCUUUGUGAGCAAUAGUAAGGAAGAGGAAA<br>AACAACAACUGUAACUAGAAGUGCUGCACGUUGUUGGCCCACGCGGGAGG<br>CCCGGGUUCGAUUCCCGGCCCAUGCACCA	58,120
TTR-siRNA	180	GCAUGGGUGGUUCAGUGGUAGAAUUCUCGCCUGGCCAGCUGUGAGUGUUU<br>CUUAAUGGAAUACUCUUGGUUACAUUGUGAGCAAUAGUAAGGAAGUGUAAC<br>CAGAGCUAUUCCAUCUAGAAGUGCUGCACGUUGUUGGCCCACGCGGGAGG<br>CCCGGGUUCGAUUCCCGGCCCAUGCACCA	58,174
ALAS1-siRNA	180	GCAUGGGUGGUUCAGUGGUAGAAUUCUCGCCUGGCCAGCUGUGAGUGUU<br>UCUUUAAGAUGAGACACUCUUUCUGGUGUGAGCAAUAGUAAGGAACCAGAG<br>AGAUGUACUCAUCUUCUAGAAGUGCUGCACGUUGUUGGCCCACGCGGGAG<br>GCCCGGGUUCGAUUCCCGGCCCAUGCACCA	58,189

Supplementary Table S2. Primer sequences used for the construction of expression plasmids.

**BioRNA**		**Cloning Primers (5' to 3')**
**BioRNA^{Gly}**	F	TTGTAACGCTGAATTCGCATGGGTGGTTCAGTGGTAGAATTCTCGCCTGGCCAGCTGTGAGTG
	R	CTTTCGCTAAGGATCTGCAGTGGTGCATGGGCCGGGAATCGAACCCGGGCCTCCCGCGTGGGCC<br>AACAACGTGC

Supplementary Table S3. Primer sequences used for stem loop RT-qPCR and RT-qPCR.

Target		Primer Sequence
GFP	Forward	5'- GCGCGCAGTTGTACTCCAGCTT-3'
	Reverse	5'- GTGCAGGGTCCGAGGT-3'
Stem loop RT		5′-GTCGTATCCAGTGCAGGGTCCGAGGTATTCGCACTGGATACGACGGGCAC-3′
BCL-2	Forward	5'- CGCGCCTACAGTTCCACAAAG -3'
	Reverse	5'- GTGCAGGGTCCGAGGT -3'
Stem loop RT		5′-GTCGTATCCAGTGCAGGGTCCGAGGTATTCGCACTGGATACGACGGATGC-3'
PD-L1	Forward	5'- CGCGCAATGCGTTCAGCAAAT -3'
	Reverse	5'- GTGCAGGGTCCGAGGT -3'
Stem loop RT		5′-GTCGTATCCAGTGCAGGGTCCGAGGTATTCGCACTGGATACGACACTGGC-3'
U6	Forward	5'-CTCGCTTCGGCAGCACA-3'
	Reverse	5'-AACGCTTCACGAATTTGCGT-3'
GFP	Forward	5′-ACGTAAACGGCCACAAGTTC-3′
	Reverse	5′-AAGTCGTGCTGCTTCATGTG-3′
BCL-2	Forward	5′-ATGCCTTTGTGGAACTGTACGGC-3′
	Reverse	5′-GATAGGCACCCAGGGTGATGC-3′
PD-L1	Forward	5'-TGCCGACTACAAGCGAATTACTG-3'
	Reverse	5'-CTGCTTGTCCAGATGACTTCGG-3'
18s	Forward	5′-AAGTCCCTGCCCTTTGTACACA-3′
	Reverse	5′-GATCCGAGGGCCTCACTAAAC-3′

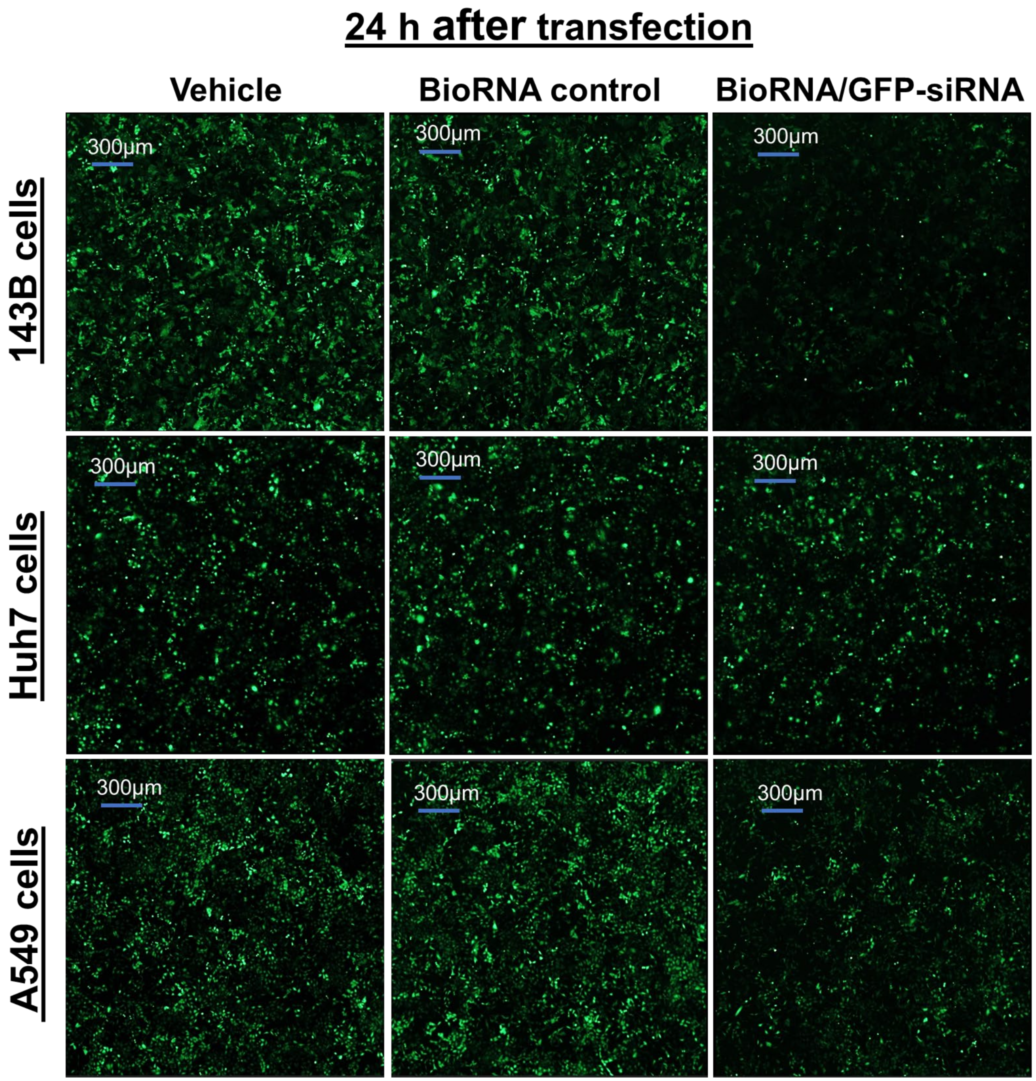

Supplemental Figure S1. GFP fluorescence intensities were reduced by recombinant GFP-siRNA in multiple cell lines after 24 h post-transfection. GFP-expressing Huh7, 143B, and A549 cells were treated with 15 nM of BioRNA/GFP-siRNA, control RNA or vehicle, and images were acquired by using ImageXpress® Pico.
